# Effect of transpulmonary pressure-guided positive end-expiratory pressure titration on lung injury in pigs with acute respiratory distress syndrome

**DOI:** 10.1007/s10877-019-00267-2

**Published:** 2019-03-22

**Authors:** Xiaoyan Wu, Ruiqiang Zheng, Zhiqing Zhuang

**Affiliations:** 1 Department of Critical Care Medicine, Clinical Medical College, YangZhou University, Northern Jiangsu People’s Hospital, Yangzhou, 225001 Jiangsu China; 2 Department of Neurology, Clinical Medical College, Wutaishan Hospital, YangZhou University, Yangzhou, 225001 Jiangsu China

**Keywords:** Transpulmonary pressure, Esophageal pressure, Positive end-expiratory pressure, Acute respiratory distress syndrome, Mechanical ventilation, Lung injury

## Abstract

To investigate the effect of positive end-expiratory pressure (PEEP) guided by transpulmonary pressure or with maximum oxygenation-directed PEEP on lung injury in a porcine model of acute respiratory distress syndrome (ARDS). The porcine model of ARDS was induced in 12 standard pigs by intratracheal infusion with normal saline. The pigs were then randomly divided into two groups who were ventilated with the lung-protective strategy of low tidal volume (VT) (6 ml/kg), using different methods to titrate PEEP level: transpulmonary pressure (TP group; n = 6) or maximum oxygenation (MO group; n = 6). Gas exchange, pulmonary mechanics, and hemodynamics were determined and pulmonary inflammatory response indices were measured after 4 h of ventilation. The titrated PEEP level in the TP group (6.12 ± 0.89 cmH_2_O) was significantly lower than that in the MO group (11.33 ± 2.07 cmH2O) (P < 0.05). The PaO_2_/FiO_2_ (P/F) after PEEP titration both improved in the TP and MO groups as compared with that at T0 (when the criteria for ARDS were obtained). The P/F in the TP group did not differ significantly from that in the MO group during the 4 h of ventilation (P > 0.05). Respiratory system compliance and lung compliance were significantly improved in the TP group compared to the MO group (P < 0.05). The VD/VT in the TP group was significantly lower than that in the MO group after 4 h of ventilation (P < 0.05). Central venous pressure increased and the cardiac index decreased significantly in the MO group as compared with the TP group (P < 0.05), whereas oxygen delivery did not differ significantly between the groups (P > 0.05). The pulmonary vascular permeability index and the extravascular lung water index in the TP group were significantly lower than those in the MO group (P < 0.05). The TP group had a lower lung wet to dry weight ratio, lung injury score, and MPO, TNF-, and IL-8 concentrations than the MO group (P < 0.05). In summary, in a pig model of ARDS, ventilation with low VT and transpulmonary pressure-guided PEEP adjustment was associated with improved compliance, reduced dead space ventilation, increased cardiac output, and relieved lung injury, as compared to maximum oxygenation-guide PEEP adjustment.

## Introduction

Although mechanical ventilation has improved survival in patients with acute respiratory distress syndrome (ARDS), it can also cause further lung injury. Indeed, ventilator-induced lung injury is widely recognized as an important contributor to morbidity and mortality in ARDS [1].

It is clearly beneficial to use low tidal volumes in patients with ARDS [2, 3]; however, it may be inappropriate to set a fixed tidal volume, regardless of the size of the ‘baby’ lungs, as this poses the risk of overdistension of the non-dependent lung [4, 5]. Positive end-expiratory pressure (PEEP) is used to prevent dependent atelectasis, but it remains unclear how to select the optimal PEEP. Several studies have shown that using increased PEEP in patients with ARDS conferred no significant clinical benefits [6, 7], even though many other studies have shown that PEEP has a protective effect [8]. The mortality rate of patients with ARDS, however, has remained high over the last two decades, despite the implementation of lung-protective ventilation. One reason for this may be that such global parameters (a fixed tidal volume, airway pressure, etc.) reflect distension of the whole lung and the chest wall, rather than the transpulmonary pressure (Ptp), the actual distending pressure of the lung [9–11]. Thus, relying on such parameters may cause overdistension of the non-dependent lung and under-recruitment of the dependent lung, thereby exacerbating lung injury.

It has been proposed that lung mechanics are a better surrogate than gas exchange for bedside assessment of lung recruitment, and that the PEEP should be selected specifically for each individually [12, 13]. Studies conducted by Talmor et al. [14] and Chiumello et al. [15] compared Ptp and the fraction of inspired oxygen (FiO_2_)-PEEP manometry in PEEP titration; they found that the Ptp-guided PEEP level was higher than that determined by FiO_2_-PEEP manometry. However, the use of maximum oxygenation manometry, another gas exchange-directed PEEP-titration method, was not investigated. We therefore conducted a randomized controlled pilot study to test the hypothesis that PEEP titration based on Ptp would relieve lung injury better than that based on maximum oxygenation in an ARDS model.

## Measurements and experimental protocol

### Animal preparation

The present animal experiment was approved by the Institutional Animal Use and Care Committee of YangZhou University, Jiangsu, China. Twelve domestic pigs, weighing 20–25 kg, were pre-anesthetized with ketamine (20 mg/kg, intramuscular, IM) and xylazine (2 mg/kg, IM),10–15 min before intubation. All animals received continuous intravenous infusion of propofol at a rate of 25–90 mg/h and remifentanil at a rate of 0.02–0.06 µg/kg/min, depending on the depth of sedation. Ringer bicarbonate solution was infused at 4 ml/kg/h, and epinephrine was occasionally administered to maintain normal blood pressure.

The animals were ventilated with a Galileo ventilator (Hamilton Medical AG, Bonaduz, Switzerland). Ventilator baseline settings in the mode of volume control (VC) were as follows: tidal volume (VT) 6 ml/kg, PEEP 5 cmH_2_O, FiO_2_ 0.5, and respiratory rate 30–40 breaths/min. Respiratory rate was adjusted to maintain arterial carbon dioxide (PaCO_2_) between 35 and 45 cmH_2_O.

Esophageal pressure (Pes) was measured with Galileo ventilator and a nasogastric esophageal balloon catheter positioned by pressure tracing (i.e. obvious cardiac inflections during expiration and a parallel rise and fall in Pes with airway pressure through the “occlusions test”), as described by Talmor and Fessler [9].

A double-lumen catheter was placed into the right internal jugular vein for fluid, anesthesia, central venous pressure (CVP) monitoring, and drug infusion. A PiCCO Pulsiocath 5 French catheter (PULSION, Feldkirchen, German) was inserted for blood gas measurements, systemic arterial blood pressure monitoring, and calculation of continuous cardiac output (CO) and extravascular lung water (EVLW).

We administered 0.9% sodium chloride intratracheally (30 ml/kg), followed by a 5-s recruitment maneuver (RM) with a PEEP of 20 cmH_2_O until the criteria for ARDS were met. ARDS was defined as oxygenation index (PaO_2_/FiO_2_) (P/F) < 100 mmHg [16].

### Lung recruitment maneuver (LRM) and PEEP titration

LRM were performed with 40 cmH_2_O PEEP for 30 s in the continuous positive airway pressure mode, followed by return to baseline ventilation, except that PEEP was maintained at 20 cmH_2_O.Arterial blood gas was measured after 5 min. LRM was repeated until P/F ≥ 400 mmHg (maximal oxygenation), or until there was < 10% difference between two consecutive measurements of P/F. This was regarded as maximized lung recruitment [17].

#### PEEP titration with maximum oxygenation

After LRM, PEEP decreased 3 cmH_2_O every 5 min from 20 cmH_2_O in a stepwise manner. Arterial blood gas was measured after 5 min. If the P/F < 400 mmHg or decreased > 10% between two consecutive measurements, the PEEP level would not decrease, and then back to the previous level after a LRM [17]. VT was set to 6 ml/kg and FiO_2_ remained at 0.5.

#### PEEP titration with Ptp

End—inspiratory Ptp (Ptp-ins), end -expiratory Ptp (Ptp-exp), respiratory system compliance (C_RS_), lung compliance (C_lung_) and chest wall compliance (C_CW_), were calculated using the standard formulae by monitoring end-expiratory and -inspiratory airway and esophageal pressure during an expiratory and an inspiratory pause of 2 s [18], as follows:

$${{\text{C}}_{{\text{RS}}}}={\text{VT}}/\left( {{\text{inspiratory Paw}}-{\text{expiratory Paw}}} \right)$$

$${{\text{C}}_{{\text{lung}}}}={\text{VT/}}\left( {{\text{inspiratory Ptp}}-{\text{expiratory Ptp}}} \right)$$

$${{\text{C}}_{{\text{CW}}}}={\text{VT}}/\left( {{\text{inspiratory Pes}}-{\text{expiratory Pes}}} \right)$$

$${\text{Ptp}} - {\text{exp}}={\text{expiratory Paw}} - {\text{expiratory Pes}}$$

$${\text{Ptp}} - {\text{ins}}={\text{inspiratory Paw}} \times \left( {{{\text{E}}_{{\text{lung}}}}{\text{/ }}{{\text{E}}_{{\text{RS}}}}} \right)$$

After LRM, PEEP decreased 3 cmH_2_O every 5 min from 20 cmH_2_O in a stepwise manner to achieve a Ptp > 0 cmH_2_O. If the Ptp < 0 cmH_2_O between two consecutive measurements, the PEEP level would not decrease, and then back to the previous level after a LRM [17]. The FiO_2_ levels were set according to a sliding scale (Table 1), based on the Ptp and FiO_2_, to keep the partial pressure of arterial oxygen (PaO_2_) between 55 and 120 mmHg [14].We also limited VT to maintain transpulmonary pressure at less than 25 cmH_2_O at the end of inspiration [19], although this limit was rarely approached, and VT (6 ml/kg) was never reduced for this purpose.

**Table 1:** Transpulmonary pressure-guided PEEP titration

FiO_2_	0.4	0.5	0.5	0.6	0.6	0.7	0.7	0.8	0.8	0.9	0.9	1.0
Ptrans-exp	0	0	2	2	4	4	6	6	8	8	10	10

Pigs with ARDS were randomly assigned to two groups of six animals each: the maximum oxygenation group (MO group) and the transpulmonary pressure group (TP group). All animals were killed with an overdose of anesthesia after 4 h of ventilation.

### Measurement of hemodynamics, gas exchange, and lung mechanics

Measurements were made immediately and repeated three times for an average within ten minutes after each time point of the baseline (T0), surfactant depletion with normal saline (NS), and every hour after randomization (T1–T4). Mean arterial blood pressure and heart rate were recorded and monitored continuously (Model 1500, Spacelabs, Issaquah, MN, USA). Cardiac preload measurements, such as the global end-diastolic volume index (GEDI) and intrathoracic blood volume index (ITBI), CO index (CI), pulmonary vascular permeability index (PVPI), and EVLW index, were monitored using pulse-induced contour cardiac output (PiCCO) technique (PiCCO *plus*, PULSION Medical Systems SE, Munich, Germany). Hemoglobin (HB) and oxygen saturation (SaO_2_) were measured to calculate the oxygen delivery (DO_2_) according to the following formula:

$${\text{D}}{{\text{O}}_2}={\text{CI}} \times \left( {1.34 \times {\text{Hb}} \times {\text{Sa}}{{\text{O}}_2}+0.0031 \times {\text{Pa}}{{\text{O}}_2}} \right) \times 10.$$

Arterial blood gases were measured using an automated blood gas analyzer (Nova, Waltham, MA, USA) and the dead space fraction (V_D_/V_T_) was measured using volumetric capnography of single respiratory CO_2_ with a NICO monitor (Novametrix Medical Systems, Inc., Wallingford, CT, USA) [20]; V_D_/V_T_ was calculated as the ratio of the difference between PaCO_2_ and the partial pressure of expiratory carbon dioxide (PeCO_2_) and PaCO_2_ (V_D_/V_T_ = (PaCO_2_–PeCO_2_)/PaCO_2_). VT, peak airway pressure (Ppeak), plat pressure (Pplat), mean airway pressure (Pmean) and PEEP were obtained from the ventilator, and Ptp was calculated as the difference between airway pressure (Paw) and Pes according the previous standard formulae [18].

### Estimation of pulmonary edema

Pulmonary edema was estimated by the ratio of the lung wet to dry weight (W/D), which is usually used to reflect the severity of pulmonary edema. Briefly, the non-dependent (lobus apicalis and cardiacus) and dependent (lobus diaphragmaticus) lung zones were removed and cleared of all extrapulmonary tissues. The lung was weighed before drying and then dried at 80 °C until the weight was constant.

### Histopathological assay

Lung injury was determined based on the findings in ten randomly selected low-power fields (× 100) for each tissue slide. Edema, alveoli and interstitial inflammation and hemorrhage, atelectasis, necrosis, and hyaline membrane formation were separately scored on a 0–4 point scale: 0 point, no injury; 1 point, injury in 25% of in the field; 2 point, injury in 50%; 3 point, injury in 75%; and 4 point, injury throughout the field. The lung injury was finally scored based on the sum of above scores.

### Markers

Markers of inflammation that are known to be increased in animal models of ALI and human ARDS were used as indicators of lung injury. Analyses for tumor necrosis factor alpha (TNF-α) and interleukin-8(IL-8) were performed in duplicate, in blinded fashion on the lung tissue homogenate of the non-dependent and dependent zones using specific enzyme-linked immunosorbent assay kits (R&D Systems, Minneapolis, MN, USA). Myeloperoxidase (MPO) activity in the lung parenchyma, used as a marker of neutrophil infiltration, was assessed by chromometry as described elsewhere [21], using a commercially available kit (Jiancheng Bioengineering Co. Nanjing, China).

### Statistical analyses

Statistical analyses were conducted using the SPSS17.0 software package (SPSS, Inc., Chicago, IL, USA). The normal distribution test was conducted in the variables. All continuous data were expressed as means ± standard deviation. Comparisons of hemodynamics, gas exchange, and lung mechanics were made between groups, using repeated-measures analysis of variance (ANOVA). One-way ANOVA was used to test for statistical differences in W/D, lung injury score, and TNF-a, IL-8 and MPO levels. A multiple comparison procedure (Student–Newman–Keuls test) was used after the ANOVA yielded significant results. A significant difference was defined as *P* < 0.05.

## Results

There were no significant differences in gas exchange, lung mechanics or hemodynamic parameters at baseline and after surfactant depletion with NS (before the randomization of animals into groups) between the two group (*P* > 0.05). After surfactant depletion, the PaO_2_/FiO_2_ was: 82.66 ± 14.46 mmHg (TP group), and 84.33 ± 20.27 mmHg (MO group), and the C_lung_ was significantly reduced from the control value by 62% ± 11% (TP group) and 71% ± 8% (MO group).

### Effect of transpulmonary pressure-guided PEEP titration on lung mechanics and gas exchange

The titrated PEEP level in the TP group (6.12 ± 0.89 cmH_2_O) was significantly lower than that in the MO group (11.33 ± 2.07 cmH_2_O) (*P* < 0.05). The Ptp-exp, Ptp-ins, Pplat and Pmean in the MO group were significantly higher than those in the TP group during the 4-h ventilation period (*P* < 0.05). C_lung_ and C_rs_ were significantly improved and were higher in the TP group than in the MO group during the 4 h of ventilation (*P* < 0.05) (Table 2).

**Table 2:** Effect of transpulmonary pressure-guided PEEP titration on lung mechanics in ARDS (n = 6, $\overline {{\upchi}}$ ± S)

	Group	T base	T0	T1	T2	T3	T4
PEEP (cmH_2_O)	TP	5.08 ± 0.12	5.12 ± 0.11	6.12 ± 0.89^▲^	6.17 ± 0.98^▲^	6.50 ± 0.55^▲^	6.00 ± 0.89^▲^
	MO	5.10 ± 0.24	5.06 ± 0.06	11.33 ± 2.07^●★^	11.26 ± 2.05^●★^	11.41 ± 2.12^●★^	11.36 ± 2.13^●★^
Pmean (cmH_2_O)	TP	7.92 ± 1.32	9.87 ± 2.38	11.06 ± 2.01^●▲^	10.63 ± 1.55^●▲^	11.10 ± 1.99^●▲^	10.83 ± 1.43^●▲^
	MO	8.13 ± 0.27	11.33 ± 2.07^●^	13.50 ± 0.55^●★^	13.67 ± 1.51^●★^	13.33 ± 1.03^●★^	13.50 ± 0.55^●★^
Pplat (cmH_2_O)	TP	14.00 ± 4.00	20.33 ± 8.02^●^	21.17 ± 5.78^●▲^	21.56 ± 3.92^●▲^	21.08 ± 4.50^●▲^	21.00 ± 6.20^●▲^
	MO	17.00 ± 4.58	22.16 ± 4.58^●^	25.67 ± 4.03^●^	25.78 ± 4.59^●^	25.97 ± 6.09^●^	26.34 ± 2.88^●^
Pes insp (cmH_2_O)	TP	6.37 ± 1.37	7.00 ± 1.03	7.97 ± 1.29	8.58 ± 0.46	8.12 ± 0.58	8.45 ± 0.90
	MO	7.33 ± 1.39	7.97 ± 1.40	9.27 ± 2.51	9.80 ± 1.70	9.93 ± 1.52	9.37 ± 1.89
Pes exp (cmH_2_O)	TP	4.83 ± 1.54	6.01 ± 0.29	6.01 ± 0.09	6.10 ± 0.21	6.23 ± 0.08	5.98 ± 0.12
	MO	5.03 ± 1.47	6.38 ± 0.54	7.36 ± 0.47	7.38 ± 0.56	7.41 ± 0.55	7.39 ± 0.52
Ptrans-exp (cmH_2_O)	TP	0.32 ± 0.06	−1.08 ± 0.32^●^	0.07 ± 0.01^●★▲^	0.18 ± 2.58^●★▲^	0.30 ± 0.14^●★▲^	0.10 ± 0.05^●★▲^
	MO	0.24 ± 0.09	−1.29 ± 0.30^●^	4.03 ± 0.63^●★^	4.06 ± 0.58^●★^	4.05 ± 0.62^●★^	4.12 ± 0.70^●★^
Ptrans-ins (cmH_2_O)	TP	9.63 ± 0.96	15.48 ± 3.12^●^	15.77 ± 2.07^●▲^	15.68 ± 2.58^●▲^	15.30 ± 0.84^●▲^	15.10 ± 2.35^●▲^
	MO	11.63 ± 2.40	17.40 ± 6.30^●^	19.93 ± 6.23^●^	20.86 ± 3.58^●^	20.88 ± 3.79^●^	19.80 ± 3.70^●^
Crs (ml/cmH_2_O)	TP	36.33 ± 12.64	15.09 ± 3.17^●^	24.44 ± 2.71^●★▲^	25.47 ± 5.53^●★▲^	25.81 ± 3.04^●★▲^	25.31 ± 5.56^●★▲^
	MO	26.53 ± 9.22	10.25 ± 4.30^●^	16.25 ± 6.48^●^	15.56 ± 5.07^●^	15.29 ± 5.85^●^	15.36 ± 4.41^●^
C_CW_ (ml/cmH_2_O)	TP	109.45 ± 19.28	74.60 ± 30.92^●^	82.27 ± 19.80	83.26 ± 47.72	85.71 ± 34.03	83.52 ± 23.03
	MO	92.69 ± 24.34	70.25 ± 6.45^●^	83.59 ± 55.34	8646 ± 45.47	87.78 ± 56.34	82.68 ± 47.87
C_L_ (ml/ cmH_2_O)	TP	52.42 ± 25.75	19.36 ± 5.04^●^	32.58 ± 2.45^●★▲^	31.23 ± 7.17^●★▲^	31.63 ± 4.29^●★▲^	33.10 ± 8.74^●★▲^
	MO	42.16 ± 24.53	12.47 ± 6.19^●^	20.42 ± 7.27^●^	18.30 ± 5.25^●^	18.25 ± 6.08^●^	20.77 ± 4.38^●^

T base: baseline state; T0: when the criteria for ARDS were obtained; T1: ventilation 1 h after randomization; T2: ventilation 2 h after randomization; T3: ventilation 1 h after randomization; T4: ventilation 4 h after randomization

^●^*P* < 0.05 as compared with T base; ^★^*P* < 0.05 as compared with T0; ^▲^*P* < 0.05 as compared with MO group at the same time point

The P/F after PEEP titration improved significantly (*P* < 0.05) in both the TP and MO group as compared with that at baseline (when the criteria for ARDS were obtained); however, the P/F did not differ significantly between the two groups during the 4-h ventilation period (*P* > 0.05). The V_D_/V_T_ in the TP group was significantly lower than that in the MO group after 4 h of ventilation (*P* < 0.05) (Table 3).

**Table 3:** Effect of transpulmonary pressure-guided PEEP titration on gas exchange in ARDS (n = 6, $\overline {{\upchi}}$ ± S)

	Group	T base	T0	T1	T2	T3	T4
PaO_2_/FiO_2_ (mmHg)	TP	461.25 ± 52.54	82.66 ± 14.46^●^	268.17 ± 148.54^●★^	270.83 ± 57.94^●★^	266.00 ± 59.70^●★^	265.67 ± 59.74^●★^
	MO	488.91 ± 62.29	84.33 ± 20.27^●^	292 ± 57.27^★^	288 ± 64.57^★^	289.83 ± 63.47^★^	287.33 ± 54.36^★^
SaO_2_ (%)	TP	99.50 ± 1.22	91.16 ± 4.40^●^	98.16 ± 2.23^★^	98.83 ± 1.47^★^	99.33 ± 0.82^★^	98.67 ± 1.37^★^
	MO	100.0 ± 0.00	92.00 ± 4.90^●^	99.33 ± 0.82^★^	99.83 ± 0.41^★^	100.0 ± 0.00^★^	99.17 ± 0.41^★^
PHa	TP	7.42 ± 0.09	7.24 ± 0.02^●^	7.35 ± 0.13	7.33 ± 0.13	7.36 ± 0.09	7.38 ± 0.07
	MO	7.44 ± 0.05	7.25 ± 0.02^●^	7.36 ± 0.14	7.36 ± 0.11	7.38 ± 0.11	7.38 ± 0.03
PaCO_2_ (mmHg)	TP	41.50 ± 4.89	53.83 ± 7.14^●^	48.40 ± 3.51^★●^	47.33 ± 1.75^★●^	48.83 ± 2.23^★●^	49.83 ± 3.65^★●^
	MO	40.50 ± 3.99	54.17 ± 7.36^●^	47.50 ± 2.74^★●^	46.50 ± 1.64^★●^	46.33 ± 4.03^★●^	49.33 ± 2.66^●^
V_D_/V_T_	TP	0.31 ± 0.02	0.59 ± 0.04^●^	0.51 ± 0.02^●★▲^	0.52 ± 0.03^●★▲^	0.51 ± 0.02^●★▲^	0.50 ± 0.01^●★▲^
	MO	0.33 ± 0.05	0.61 ± 0.05^●^	0.58 ± 0.06^●^	0.59 ± 0.04^●^	0.59 ± 0.04^●^	0.60 ± 0.03^●^

^●^*P* < 0.05 as compared with T base; ^★^*P* < 0.05 as compared with T0; ^▲^*P* < 0.05 as compared with MO group at the same time point

### Effect of transpulmonary pressure-guided PEEP titration on hemodynamic

The CVP increased and CI decreased significantly in the MO group as compared to the TP group (*P* < 0.05), whereas the DO_2_ did not significantly differ between the two groups during the 4 h ventilation after PEEP titration (*P* > 0.05). PVPI and EVLWI in the TP group were markedly decreased as compared to those at baseline (*P* < 0.05) and were significantly lower than those of the MO group at T1-4 (*P* < 0.05) (Table 4).

**Table 4:** Effect of transpulmonary pressure-directed PEEP titration on hemodynamics in ARDS (n = 6, $\overline {{\upchi}}$ ± S)

	Group	T base	T0	T1	T2	T3	T4
HR (beat/min)	TP	89.83 ± 10.38	115.33 ± 19.95^●^	116.50 ± 16.42 ^●^	116.00 ± 14.71 ^●^	118.17 ± 11.61 ^●^	120.67 ± 10.34 ^●^
	MO	93.33 ± 10.80	110.67 ± 21.50 ^●^	114.83 ± 12.77 ^●^	120.33 ± 17.52 ^●^	124.0 ± 14.91 ^●^	127.83 ± 13.09 ^●^
MAP (mmHg)	TP	120.33 ± 17.41	107.16 ± 17.92	111.00 ± 11.56	111.17 ± 9.20	105.83 ± 14.02	101.33 ± 14.18^●^
	MO	120.17 ± 10.93	106.33 ± 12.27	105.83 ± 25.13	105.17 ± 27.45	98.67 ± 21.10^●^	89.17 ± 13.89^●^
CVP (mmHg)	TP	5.73 ± 0.65	6.02 ± 0.89	6.33 ± 0.82^▲^	6.35 ± 1.21^▲^	6.33 ± 1.11^▲^	6.50 ± 1.05^▲^
	MO	5.83 ± 0.75	6.00 ± 0.63	9.17 ± 0.75^●★^	9.17 ± 0.98^●★^	9.33 ± 0.81^●★^	9.33 ± 0.81^●★^
CI (l/min/m^2^)	TP	4.50 ± 0.29	4.49 ± 0.41	4.28 ± 0.60^▲^	4.38 ± 0.38^▲^	4.23 ± 0.30^▲^	4.42 ± 0.47^▲^
	MO	4.53 ± 0.28	4.40 ± 0.48	3.60 ± 0.42^●★^	3.34 ± 0.65^●★^	3.61 ± 0.85^●★^	3.69 ± 0.69^●★^
SVI (ml/m^2^)	TP	50.99 ± 9.79	39.78 ± 6.55	37.08 ± 5.84 ^▲^	38.49 ± 7.59^▲^	36.30 ± 6.42^●▲^	36.97 ± 6.09^●▲^
	MO	49.05 ± 5.90	40.54 ± 5.31	31.31 ± 3.99^●^	28.24 ± 4.02^●★^	29.60 ± 3.97^●★^	29.20 ± 4.66^●★^
SVRI (dyn s m^2^/cm^5^)	TP	2111.17 ± 155.38	1859.67 ± 174.29	2037.67 ± 273.70	2041.17 ± 561.78	1987.33 ± 710.05	1774.33 ± 888.10
	MO	2079.00 ± 228.29	1856.00 ± 127.58	2233.00 ± 419.28	2392.33 ± 562.29	2108.33 ± 466.10	1789.67 ± 476.69
dPmax (mmHg /s)	TP	773.5 ± 61.15	737.67 ± 111.27	766.83 ± 90.02	707.33 ± 58.39	775.0 ± 127.36	799.17 ± 166.02
	MO	827.50 ± 117.38	760.50 ± 48.12	753.0 ± 47.00	726.67 ± 38.17	733.17 ± 19.66	733.17 ± 25.30
GEDI (ml/m^2^)	TP	585.50 ± 65.81	630.17 ± 124.58	604.0 ± 121.10	606.50 ± 98.83	602.33 ± 99.82	573.0 ± 89.81
	MO	594.33 ± 66.28	613.0 ± 77.32	515.67 ± 66.59	515.83 ± 78.63	518.0 ± 92.61	502.67 ± 72.40
ITBI (ml/m^2^)	TP	722.83 ± 79.72	774.50 ± 164.09	763.0 ± 146.15	749.5 ± 121.56	748.17 ± 123.16	702.0 ± 110.60
	MO	798.17 ± 71.99	743.83 ± 113.64	653.50 ± 104.12	658.50 ± 126.99	659.0 ± 150.65	657.5 ± 106.69
LAC (mmol/l)	TP	1.46 ± 0.23	3.03 ± 0.51^●^	2.65 ± 0.45	2.15 ± 0.34	1.83 ± 0.39^★^	1.65 ± 0.28^★^
	MO	1.53 ± 0.23	2.95 ± 0.40^●^	2.57 ± 0.45	2.13 ± 0.48	1.80 ± 0.25^★^	1.63 ± 0.18^★^
DO2 (ml/min/m_2_)	TP	485.57 ± 46.17	321.81 ± 8.29^●^	464.74 ± 97.34^★^	504.18 ± 119.35^★^	506.86 ± 146.13^★^	485.46 ± 126.51^★^
	MO	473.13 ± 38.90	320.16 ± 16.42^●^	505.33 ± 71.89^★^	513.05 ± 38.81^★^	481.13 ± 59.30^★^	537.32 ± 92.19^★^
Fluid infusion (ml/kg)	TP	–	–	4.5 ± 0.4	4.4 ± 0.3	4.2 ± 0.4	4.6 ± 0.5
	MO	–	–	5.1 ± 0.5	4.9 ± 0.6	4.9 ± 0.5	5.0 ± 0.6
PVPI	TP	2.48 ± 0.33	5.17 ± 1.43^●^	3.77 ± 1.77^★▲^	3.88 ± 1.53^★▲^	3.65 ± 1.07^★▲^	3.52 ± 0.91^★▲^
	MO	2.35 ± 0.38	4.65 ± 0.73^●^	5.22 ± 0.96^●^	4.95 ± 1.09^●^	5.22 ± 0.96^●^	5.52 ± 0.53^●^
EVLWI (ml/kg)	TP	15.65 ± 1.33	27.5 ± 5.05^●^	18.00 ± 3.16^★▲^	18.67 ± 5.50^★▲^	18.17 ± 4.26^★▲^	17.67 ± 4.46^★▲^
	MO	15.83 ± 1.72	30.33 ± 2.87^●^	27.33 ± 3.67^●^	27.33 ± 3.67^●^	29.00 ± 2.61^●^	25.17 ± 3.49^●^

^●^*P* < 0.05 as compared with T base; ^★^*P* < 0.05 as compared with T0; ^▲^*P* < 0.05 as compared with MO group at the same time point

### Effect of transpulmonary pressure-guided PEEP titration on pulmonary edema, pathological changes and inflammatory mediators

The TP group had a lower W/D and a lung injury score in both the non-dependent and dependent lung zones than the MO group (*P* < 0.05). MPO, TNF-α, and IL-8 concentrations in the non-dependent and the dependent lung zones in the TP group decreased significantly as compared with those in the MO group after 4 h of ventilation (*P* < 0.05) (Table 5).

**Table 5:** Effect of transpulmonary pressure-guided PEEP titration on pulmonary edema, pathological changes and inflammatory mediators ARDS (n = 6, $\overline {{\upchi}}$ ± S)

	Undependent lung regions		Dependent lung regions	Total
	Lobus apicalis	Lobus cardiacus	Lobus diaphragmaticus	
W/D				
TP	4.18 ± 0.40	4.99 ± 0.73^▲^	8.15 ± 0.27	5.77 ± 0.36^▲^
MO	6.18 ± 0.34	7.49 ± 0.65	8.27 ± 0.74	7.31 ± 0.58
Lung injury score				
TP	2.25 ± 0.56^▲^	3.63 ± 0.35^▲^	5.92 ± 1.29	3.93 ± 0.63^▲^
MO	3.51 ± 0.26	5.32 ± 0.84	7.42 ± 0.16	5.42 ± 0.25
MPO (U/g)				
TP	1.23 ± 0.16^▲^	1.40 ± 0.64	2.67 ± 0.46	1.76 ± 0.27 ^▲^
MO	2.25 ± 0.26	2.45 ± 0.55	2.73 ± 0.23	2.48 ± 0.23
TNF-α (pg/ml)				
TP	256 ± 88^▲^	476 ± 90^▲^	602 ± 123	445 ± 92^▲^
MO	458 ± 92	756 ± 92	898 ± 92	704 ± 92
IL-8 (pg/ml)				
TP	112.9 ± 24.2^▲^	123.5 ± 30.2^▲^	166.4 ± 30.1`	134.9 ± 26.2^▲^
MO	192.8 ± 24.4	222.8 ± 44.7	256.8 ± 35.8	223.8 ± 35.4

^▲^*P* < 0.05 as compared with MO group

## Discussion

In a porcine model of ARDS ventilated with low VT and with Ptp at end- inspiration limited to 25 cmH_2_O, Ptp-guided PEEP adjustment was associated with improved compliance, reduced dead space ventilation, increased CO, and relieved lung injury, as compared to optimal oxygenation-guided PEEP adjustment. However, the approach used to guide PEEP adjustment had no significant effect on oxygenation and oxygenation transport.

The lung-protective strategy of limiting VT and using PEEP has been demonstrated to improve the prognosis of ARDS patients [2, 3, 6–8]. Even with adoption of the low VT ventilation strategy, tidal alveoli may suffer hyperinflation [4, 5], mostly in non-dependent lung regions, and consequently increasing Ptp at inspiration may augment lung injury. If pleural pressures are similarly variable, a given level of PEEP could be inadequately low in patients with high pleural pressure or dangerously high in patients with a low pleural pressure. Failure to take into account differing levels of pleural pressure could confound attempts to determine optimum PEEP in patients with ARDS, which may lead to overdistension of alveoli or dependent atelectasis and inspiratory stretch. Yoshida et al. [22] showed that expiratory Ptp derived from Pes reflects the Ptp in the dependent to middle lung sections, where atelectasis usually predominates; inspiratory Ptp estimated from the elastance ratio may indicate the highest level of lung stress in the non-dependent lung.

Traditionally, Pes has been used as a surrogate of pleural pressure in respiratory physiology, although it has been reported that there are discrepancies between these measurements [23]. Factors such as lung volume, compression by the mediastinum, and the pleural recording site may explain these differences [23, 24]. To overcome this problem, it has been suggested that a correction should be made in Ptp calculation, adding an arbitrary value of cmH_2_O based on changes observed in Pes after body position modification in healthy subjects [24, 25]. We did not perform such correction in our calculations because we considered that our esophageal recording might estimate pleural pressure between the middle and the more dependent lung regions through the occlusion test, as has been suggested in an experimental ARDS model [26]. A recent paper by Yoshida et al. [22] showed that esophageal pressure (inspiratory or expiratory) could accurately reflect pleural pressure in the lung adjacent to the esophageal balloon (i.e., dependent to mid-lung region) and supported the use of esophageal manometry in ARDS.

The ratio of benefit to harm from PEEP depends on the amount of lung that can be recruited by raising PEEP, which varies widely among patients with ARDS [27]. Determination of the alveolar recruitment in ARDS is often performed by analyzing oxygenation. Excessive pursuit of maximum oxygenation often leads to excessive expansion of the lung. As shown in our study, oxygenation seemed to be improved in the MO group as compared to the TP group, due to the higher PEEP level in the former group. However, the lung compliance reduced and the dead-space fraction increased, which indicated overdistension of the alveoli. Another disadvantage of using arterial oxygenation to determine the recruitment effects is the trouble and expense of drawing and analyzing a series of arterial blood samples in a timely manner. It is well known that better oxygenation does not imply improved outcomes [7, 28]. However, catheter location, balloon volume, and correct calculation are essential for Ptp monitoring, and require experience and skill.

Various studies have reported that increased dead-space is one of the hallmarks of early ARDS, and an elevated V_D_/V_T_ is independently associated with an increased risk of death [29, 30]. Elevated V_D_/V_T_ probably reflects alterations in the distribution of pulmonary blood flow. A study by Gattinoni et al. [27] has showed that different PEEP levels after the RM caused significant changes in V_D_/V_T_. However, a higher PEEP in the optimal oxygenation-guided PEEP group in that study could increase V_D_/V_T_ by regional overdistention of well-ventilated alveoli or by reduction in CO.

Excessively high PEEP can obstruct venous return, thereby decreasing CO and necessitating greater fluid infusion to maintain blood pressure. Notably, DO_2_ did not significantly differ between the two groups. Therefore, any decrease in CO may have been offset by a slight increase in oxygen-carrying capacity. Furthermore, it is possible that the occurrence of pulmonary edema in the optimal oxygenation-guided PEEP group was unrelated to the pigs’ volume status, because excessive expansion of the alveoli can increase pulmonary permeability.

Data from various studies supported lung compliance as a marker of alveolar recruitment [31]. Whether PEEP increases or decreases compliance depends on the relative contributions of recruitment of atelectatic areas and of overdistention of alveoli. Studies in patients with ARDS reported the lung compliance and the dead-space fraction can identify an optimal PEEP level because the highest compliance value in conjunction with the lowest dead-space fraction indicates a maximum amount of effectively expanded alveoli [32]. This gain in the efficiency of ventilation implies an increase in the number of functional lung units and not a mere expansion of previously open alveoli, thereby limiting any significant overdistension. In our study, the improved compliance and decreased dead-space produced by Ptp-guided PEEP adjustment should coincide with optimum lung function. The marked decrease in compliance and increased dead space fraction at optimal oxygenation-guided PEEP group can be explained by an overdistension of already open alveoli. In agreement with our observations, in a previous study of patients with ALI, with the increase of PEEP level from 0 to 30 cmH_2_O, respiratory system mechanics and Vd/Vt did not improved, but instead worsened when the PEEP level was too high [20] .

Injury to the alveolar epithelial-endothelial barrier causes a series of intracellular signaling events that may result in the production of inflammatory mediators [33, 34]. The relevance of cytokines in ARDS has been evident from human studies, which showed an increase in bronchoalveolar lavage IL-8 concentrations that preceded clinical evidence of disease, and that bronchoalveolar lavage concentrations of TNF-α and IL-8 were higher at presentation in patients who died of ARDS than in those who survived [35, 36]. Our study found that Ptp-guided PEEP titration was associated with strikingly lower level of TNF-α and IL-8 in the non-dependent and dependent regions than observed with maximum oxygenation-guided PEEP titration, which showed that Ptp-guided PEEP titration had a greater lung-protective impact.

Infiltration of polymorphonuclear leukocyte plays a central role in the development of ARDS and MPO activity in the lung parenchyma was used as a marker of neutrophil infiltration. The finding that Ptp-guided PEEP titration markedly decreased MPO, as compared with maximum oxygenation-guided PEEP titration, further proved that Ptp-guided PEEP titration could lessen inflammatory cell infiltration and the inflammatory response.

This study had some limitations. Our study was carried out in a porcine pulmonary ARDS model, and thus our findings may not be generalizable to other ARDS etiologies. Second, in contrast with the study of Talmor et al. [10], which used comparison with ARDSnet-guided PEEP titration, Ptp-guidance resulted in a higher PEEP level. The results of comparison with other methods used to titrate the optimal PEEP, such as the maximum compliance method, minimum dead space method, etc., require further study. Third, not knowing which PEEP level each measurement reflects, so we might miss the optimal PEEP level, and PEEP need to be adjusted to the previous level before the oxygenation or trans-pulmonary pressure decreased. Finally, measurements were performed during derecruitment, and thus the mechanical behavior was influenced by previous recruitment history. Thus, our experiment may be different from those expected during an incremental PEEP titration maneuver. In summary, this randomized controlled pilot trial showed that compared with the maximum oxygenation strategy, individualized PEEP selection based on Ptp in a porcine of model of ARDS, treated with low VT and limited plateau pressure, was associated with improved compliance, reduced dead- space ventilation, increased CO, and relieved lung injury.
